# In Silico Identification of 1-DTP Inhibitors of *Corynebacterium diphtheriae* Using Phytochemicals from *Andrographis paniculata*

**DOI:** 10.3390/molecules28020909

**Published:** 2023-01-16

**Authors:** Jameel M. Al-Khayri, Sakshi Dubey, Gopishankar Thirumoorthy, Praveen Nagella, Adel Abdel-Sabour Rezk, Wael Fathi Shehata

**Affiliations:** 1 Department of Plant Biotechnology, College of Agriculture and Food Sciences, King Faisal University, Al-Ahsa 31982, Saudi Arabia; 2 Department of Life Sciences, CHRIST (Deemed to Be University), Bengaluru 560029, Karnataka, India; 3 Virus & Phytoplasma Research Department, Plant Pathology Research Institute, Agricultural Research Center, Giza 3725005, Egypt

**Keywords:** *Andrographis paniculata* (Burm. f.) Wall. ex Nees, diphtheria toxin, autodock, bisandrographolide, andrographiside and phytochemicals

## Abstract

A number of phytochemicals have been identified as promising drug molecules against a variety of diseases using an in-silico approach. The current research uses this approach to identify the phyto-derived drugs from *Andrographis paniculata* (Burm. f.) Wall. ex Nees (AP) for the treatment of diphtheria. In the present study, 18 bioactive molecules from *Andrographis paniculata* (obtained from the PubChem database) were docked against the diphtheria toxin using the AutoDock vina tool. Visualization of the top four molecules with the best dockscore, namely bisandrographolide (−10.4), andrographiside (−9.5), isoandrographolide (−9.4), and neoandrographolide (−9.1), helps gain a better understanding of the molecular interactions. Further screening using molecular dynamics simulation studies led to the identification of bisandrographolide and andrographiside as hit compounds. Investigation of pharmacokinetic properties, mainly ADMET, along with Lipinski’s rule and binding affinity considerations, narrowed down the search for a potent drug to bisandrographolide, which was the only molecule to be negative for AMES toxicity. Thus, further modification of this compound followed by in vitro and in vivo studies can be used to examine itseffectiveness against diphtheria.

## 1. Introduction

Infectious diseases with high morbidity and mortality are one of the leading causes of global disease burden, especially in developing countries. Re-emergence of these diseases is another challenge caused due to microbial adaptability and the development of antimicrobial resistance [1]. This occurs because of overuse, underuse, or misuse of antibiotics [2]. The rising prevalence of such diseases in the population necessitates the development of more powerful drugs to combat them. The amalgamation of computational and experimental methods helps to identify promising compounds against a disease. This, however, requires a huge investment of time as well as money. One way to speed up the drug discovery and development process is the computer-Aided Drug Discovery method (CADD), which can screen a large number of compounds and pick one potent molecule against a specific target [3]. The integration of high-throughput X-ray crystallography and structural genomics has paved [4,5] the way for structure-based drug design (SBDD) to become a more important part of the drug discovery process as well as modern medicinal chemistry [6].

SBDD uses virtual screening and molecular modeling to identify potent drug candidates from the chemical scaffold, which are available in various databases.Their biological characteristics are then assessed, including their affinity and efficacy, and the ligand-receptor complexes are visualized to show their binding sites and molecular interactions. The lead molecules are then subjected to ADMET and Pharmacokinetic analysis which helps in eliminating the undesirable properties; further optimization with active drug-like and lead-like properties allows these molecules to be efficiently used against the target protein [7,8]. This in silicoapproach has been used for predicting phytochemicals as promising drug molecules against several diseases, these include inhibition of angiogenesis by indigocarpan (*Indigofera aspalathoides*) [9], inhibition of main protease of SARS-COV-2 (Mpro) by andrographolide (*Andrographis paniculata*) [10], and reduction in the activity of glycogen phosphorylase by magnoflorine, cordiofolioside A, and syringin (*Tinospora cordifolia*) [11]. SBDD therefore can be used as a promising tool for predicting herbal medications against drug-resistant bacteria. One such disease is diphtheria, which is caused by *Corynebacterium diphtheriae* belonging to the family Actinomycetales. This organism majorly affects the upper respiratory tract by adhering to the cells of the pharynx, tonsils, and trachea [12,13] where it multiplies to form a pseudo membrane that can block airflow leading to suffocation and death [14], the organism also releases a toxin which causes fatal damage to heart and kidneys. This toxin is encoded by the tox gene of corynephage beta, which is a lysogenic bacteriophage carried by the causative organism [15].

Diphtheria toxin is a 560 amino acids-long polypeptide chain [16] consisting of a catalytic (C), transmembrane (T), and a receptor-binding (R) domain. The T domain aids the passage of the C domain through the membrane and into the cytosol. Here, it blocks protein synthesis by attaching an ADP-ribosyl group from NAD to a modified histidine residue of the eukaryotic ribosomal elongation factor EF2 [17,18,19]. The number of reported diphtheria cases has leveled up from 2006 to 2013 to about 4300–5700 [20], with over 41,672 cases between 2005 and 2014. India is considered a hotspot for the disease and has also witnessed several outbreaks in recent years. This can be attributed to the temperate climate that provides a conducive environment for the growth of diphtheria; due to insufficient vaccination rates and weakening vaccine immunity in adults, diphtheria has resurfaced in several countries [21]. Therefore, proper immunization against the toxigenic strains of diphtheria and broad-spectrum antibiotics, mainly penicillin and erythromycin, are crucial in the treatment of the disease [22,23].

Vancomycin, clindamycin, tetracycline, rifampin, kanamycin, and gentamicin are some of the antibiotics that can be used as alternative therapy. These antibiotics, however, target the bacteria rather than the toxin, which evades treatment and poses a serious danger to the host. Moreover, it becomes less effective as *C. diphtheriae* have the ability to harbor integrons that aid in the expression of drug resistance gene cassettes [24,25]. This necessitates the use of a non-synthetic drug molecule that shows minimalsideeffects and can perform a dual function—that is, counteract drug resistance and inhibit the activity of diphtheria toxin. Taking medicinal plants as therapeutic strategies has several benefits that include their holistic approach. It integrates a person’s mental, emotional and spiritual well-being, exhibiting a synergistic action on the physiological system as well as strengthening the body’s natural healing mechanism [26].

Pharmaceutical drugs of plant origin are found to be effective against various conditions including Alzheimer’s disease [27], cancer [28], dementia [29], diabetes [30], and cardiovascular disease [31]. Worldwide, about 80,000 species of higher plants have been identified as possessing medicinal properties. Of these, nearly 45,000 are found in India, making it one of the 12 most biodiverseregionsinthe world. Pharmaceutical drugs of plant origin have been mentioned in the ancient systems of medicine such as Ayurveda and Unani and have been used by various traditional communities [32]. One such herb is *Andrographis paniculata* (AP). Commonly known as ‘Kalmegh’ or ‘King of bitters’, this plant belongs to the family Acanthaceae and is found to exhibit a wide range of pharmacological activities [33]. These include, anti-cancerous [34], anti-inflammatory [35], anti-hyperglycemic [36], antimalarial and immunostimulatory and hepatoprotective activities [37]. The Phyto derivatives, such as diterpenoids, flavonoids, and polyphenols, are responsible for these characteristics [38]. The major active constituents of AP include 7-methylwogonin, apigenin, luteolin, andrographidine C, andrographolide, neoandrographolide, andrograpanin, onysilin, bisandrographolide, 14-Deoxy-12-hydroxyandrographolide, Andrographolactone, 3-O-beta-D-glucopyranosyl 14,19-dideoxyandrographolide, 8,17-Epoxy-14-deoxyandrographolide, 12-Hydroxyandrographolide, andisoandrographolide. The lack of information in the literature on these molecules’ unique antibacterial properties allows the scope to delve more into the nature of these phytocompounds.

This study aims to identify a potent bioactive agent against diphtheria toxin by screening 18 different phytoconstituents of AP with the catalytic domain of the toxin. An insight into the nature of molecular interactions, drug-likeness, and ADME properties helps to assess the efficacy of these bioactives, which are crucial in managing diphtheria.

## 2. Results and Discussions

### 2.1. Molecular Docking Analysis

Diphtheria toxin causes cell intoxication by interacting with the membrane receptor pro-HB-EGF, which leads to internalization and protein synthesis inhibition [39], thus making it a primary target for developing potential therapeutics. Molecular docking analysis was, therefore, done to establish the molecular interactions between the target protein and 18 different ligands. These phytochemicals were docked with the catalytic domain of diphtheria toxin by using the AutoDock Vina tool. The binding pocket of the toxin has four major amino acids namely, His21, Tyr54, Tyr65, and Glu148, which are responsible for their toxic activities [40]. 1DTP was found to show effective binding interactions with negative values of free energy in the grid box (ranging from −7.6 to −10.4) indicating a strong affinity between the two components as binding energy is inversely proportional to the activity of a compound and is known to have a strong influence on the involvement of the ligand and even the flexibility of the protein [41]. A few other compounds, including cyanidin, 3-hydroxyflavone, and 6-gingerol, have also been demonstrated to exhibit strong binding affinities with diphtheria toxin [42]. The list of active molecules and their respective free energies obtained from docking studies is shown in Table 1, with ampicillin as the control drug.

**Figure 1:**
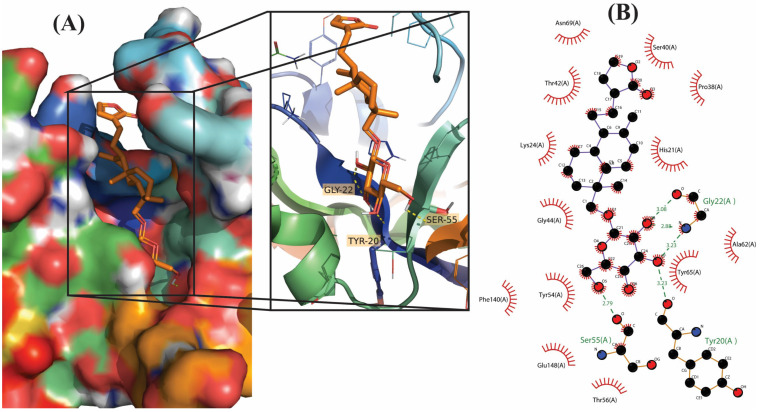
Molecular docking results of Neoandrographolide into the catalytic domain of diphtheria toxin. (**A**) Three-dimensional binding mode of Neoandrographolide in the protein active site. (**B**) Two-dimensional interaction showing amino acid residues involved in hydrogen, hydrophobic, and electrostatic interactions.

**Figure 2:**
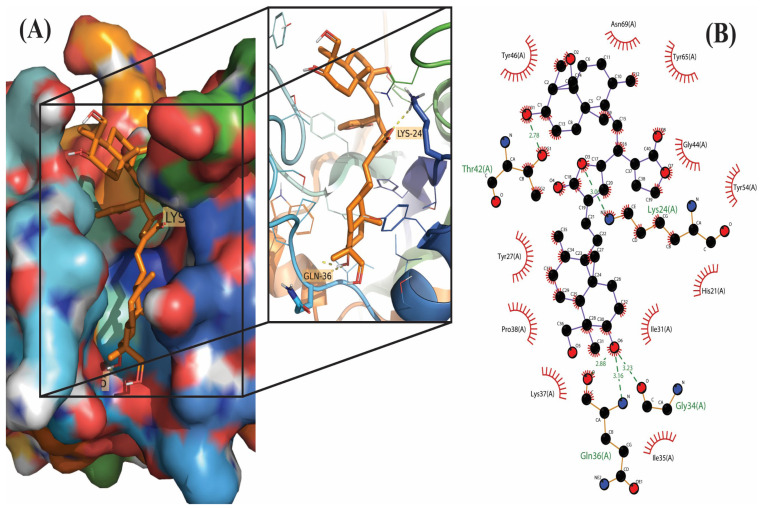
Molecular docking results of Bisandrographolide into the catalytic domain of diphtheria toxin. (**A**) Three-dimensional binding mode of Bisandrographolide in the protein active site. (**B**) Two-dimensional interaction showing amino acid residues involved in hydrogen, hydrophobic, and electrostatic interactions.

**Figure 3:**
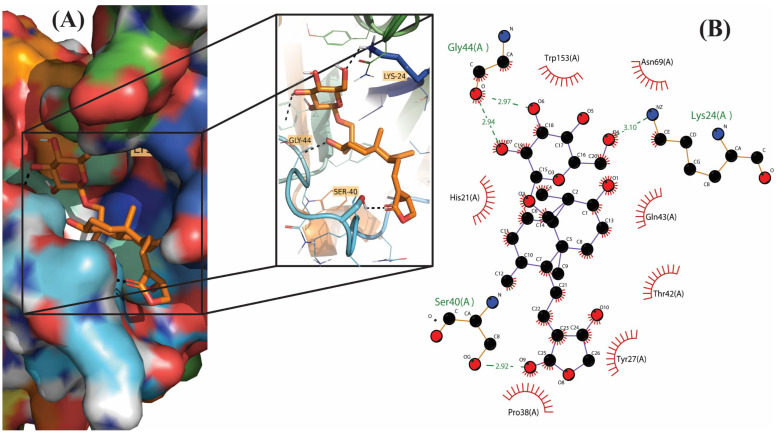
Molecular docking results of Andrographiside into the catalytic domain of diphtheria toxin. (**A**) Three-dimensional binding mode of Andrographiside in the protein active site. (**B**) Two-dimensional interaction showing amino acid residues involved in hydrogen and hydrophobic interactions.

**Figure 4:**
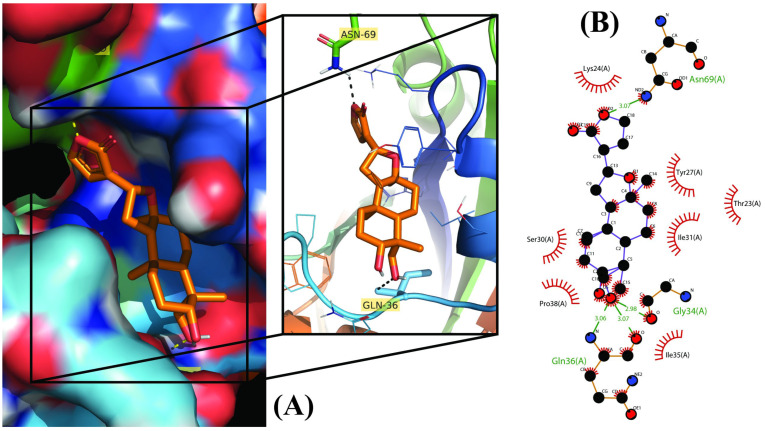
Molecular docking results of Isoandrographolide into the catalytic domain of diphtheria toxin. (**A**) Three-dimensional binding mode of Isoandrographolide in the protein active site. (**B**) Two-dimensional interactions showing amino acid residues involved in hydrogen bonding, salt-bridge, and hydrophobic interactions.

Out of 18 phytoconstituents, the binding energies of 17compounds were found to be less than the control drug (Ampicillin), which has a dockscore of −7.4. This means that the bioactive molecules of *A. paniculata* have a higher activity than the standard drug and a tendency to interact with diphtheria toxin and interfere with its activity. Four ligands having a binding affinity of−9.1 or less (Neoandrographolide, Bisandrographolide, Andrographiside, and Isoandrographolide) were chosen as the best ligands and used to analyze molecular interactions and pharmacokinetic properties (Figure 1, Figure 2, Figure 3 and Figure 4). Of these, the diterpenoid neoandrographolide is also shown to act as an antisecretory molecule against diarrhea caused by *Escherichia coli* enterotoxin [43]. They also target the fusion and adsorption of viruses such as HIV and influenza A to the host [44]. Bisandrographolide and andrographolide exhibit significant interaction with Zika virus NS2B-NS3 protease, therefore, functioning as antiviral phytopharmaceuticals [45].

### 2.2. Molecular Interactions

The molecular interactions between the ligands and receptor were visualized using the Discovery studio visualizer. The binding of compounds in the active site of the toxin were majorly operated by polar interactions along with hydrogen bonding and hydrophobic interactions. The binding orientations of the top four phytochemical analogs, namely, bisandrographolide, andrographiside, neoandrographolide, and isoandrographolide were analyzed in depth. The optimal poses were discovered, describing in detail the different amino acid residues involved in the interaction (Table 2).

It was revealed by the 3D structures of bisandrographolide and isoandrographolide that hydrophobic interactions were more prevalent in the binding pocket of the protein compared to hydrogen bonds. This probably reflects the role of these bonds in making the compound an efficient ligand [46]. In biological complexes, hydrogen bonds are the most common directional intermolecular interactions [47], and these were found to be more in the case of andrographiside and neoandrographolide. The third important interactions were the salt bridge interactions found only in bisandrographolide and isoandrographolide. This might be due to the lesser contribution of these forces toward protein stability [48,49].

Among all the phytocompounds, bisandrographolide shows the strongest interaction with diphtheria toxin, as is evident by its binding energy, which is −10.4. Three polar residues, namely, Gln (2.29 Å), Thr (2.09 Å), and Asn (2.66 Å) are involved in hydrogen bonding between the receptor and ligand whereas Tyr (3.68 Å), Ile (3.57 Å), Pro (3.69 Å), Thr (3.98 Å), and Tyr (3.66 Å) are involved in hydrophobic interaction. Conversely, three amino acids participate in electrostatic interaction, these are His (4.12 Å), His (5.12 Å), and Lys (5.12 Å). Similarly, two Lys residues [Lys (4.88 Å) and Lys (2.86 Å)] and one His residue [His (5.35 Å)] participate in salt bridge interaction between the receptor and isoandrographolide molecule, which is the third best analog with a binding affinity of −9.4; by involving amino acid residues primarily Gln (2.09 Å), Asn (3.49 Å) and Tyr (3.58 Å), Ile (3.31 Å), Pro (3.64 Å), Trp (3.66 Å), this molecule also includes hydrogen and hydrophobic interactions.

Andrographiside is the second-best drug with −9.5 as the binding energy. A detailed analysis of this molecule reveals that it fits into the active binding site of the receptor stably by interacting with the key amino acid residues namely Lys (2.38 Å), Lys (2.89 Å), Ser (2.93 Å), Ser (2.15 Å), Gly (2.75 Å), and Gly (2.44 Å), which play an important role in hydrogen bonding with the receptor, and Tyr (3.75 Å), Pro (3.33 Å), Thr (3.89), and Trp (3.72 Å), which are involved in hydrophobic interaction.

Neoandrographolide, which is the fourth-best molecule, correlates with the diphtheria toxin by forming five hydrogen bonds and a single hydrophobic interaction. The amino acids primarily involved in these interactions are Tyr (2.68 Å), His (2.74 Å), Gly (1.88 Å), Ser (1.98 Å), Ser (2.41 Å), and Tyr (3.82 Å).

### 2.3. Pharmacodynamic Analysis and Toxicity Studies

Lipinski’s rule of five has been designed to check the drug-likeness of new molecular entities [50], and, therefore, was used to evaluate the compounds’ drug-like properties using the Swiss ADME tool (http://www.swissadme.ch/ accessed on 16 June 2021). The parameters considered include the number of hydrogen bond acceptors and donors, which was found to be ≤10 and ≤5, respectively, for all four compounds; Log P and TPSA values, being important indicators of good bioavailability [51,52], were also studied and were found to range between 2.67 and 4.5 for log P and <140 for TPSA. Log p or lipophilicity values <5 is an indicator of their ability to penetrate the biological membranes [9]. Conversely, topological surface area on the other hand, defined as the sum of polar surfaces containing oxygen, nitrogen, and hydrogen atoms, is found to be associated with the hydrogen bonding potential of a compound [53].Andrographiside deviates in terms of TPSA, with a value of 166.14, as does bisandrographolide, which differs in terms of AMR (Molar refractivity) with a value of 185.52 (while other molecules fall within the range of 93.54 to 127.6) and also violates Lipinski’s rule by having a molecular weight greater than 500 (664.87 for bisandrographolide and 512.59 for andrographiside). Although a higher molecular weight indicates increased bulkiness of the molecule, which ultimately affects its permeability [54], it does not significantly classify the molecules based on their bioavailability. Furthermore, the violation of Lipinski’s rule in two or fewer parameters is acceptable [55]. In addition to this, the number of rotatable bonds was also considered and was found to be ≤10 for all the phytochemical analogs. This means that the compounds are flexible and, thus, can interact with the rigid binding site of the protein [56] (Table 3).

The major ADMET properties with different permeability i.e., BBB, CaCO_2_ permeability, human intestinal absorption, and AMES toxicity test were evaluated for the best four molecules with the aid of pkCSM and their results are incorporated in Table 4.

The results report that only bisandrographolide shows the absence of AMES toxicity, while the other three molecules were positive for this test, indicating their mutagenic capacity. In addition, bisandrographolide does not show hepatotoxicity, which indicates its non-involvement in disrupted liver function and is not an inhibitor of hERG. The BBB permeability of bisandrographolide was found to be −1.24, which means that it is poorly distributed to the brain. The maximum tolerated dose of a drug should ideally be less than or equal to 0.477 (log mg/kg/day), and for bisandrographolide was found to be −0.191. Another important aspect in the metabolism of a drug is the Cytochrome P450 or CYP enzyme, which is involved in the oxidation of xenobiotics. It is the inhibition or activation of this enzyme that determines whether a drug will be accumulated or easily excreted from the system [57]. The phytochemical under investigation is found to be a non-inhibitor of all CYP isozymes, although it does inhibit CYP3A4, necessitating further development before it can be used as an active diphtheria treatment.

### 2.4. Molecular Dynamics Simulation Studies

The MD simulation trajectories obtained after a 100 ns run helps to analyze the root means square deviation (RMSD), root means square fluctuation (RMSF), radius of gyration (RG), and hydrogen bonds. It also gives an insight into the binding energy of the protein-ligand complex throughout the MD simulation, which is calculated using the MM PBSA method.

**Figure 5:**
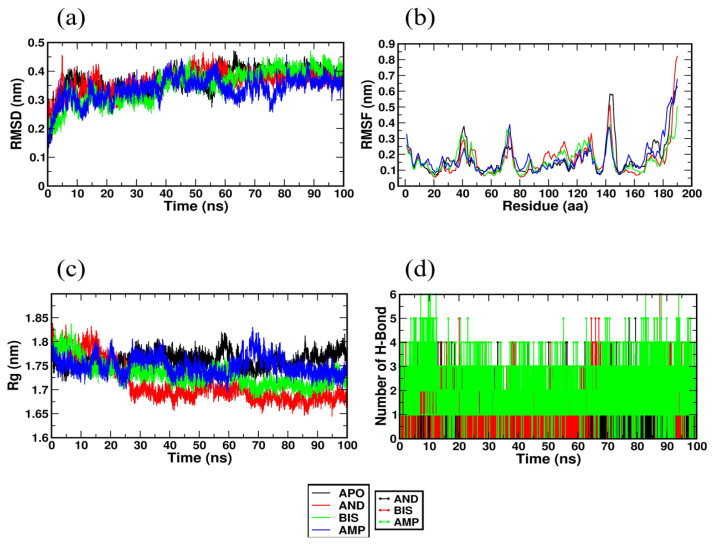
(**a**) Root means square deviation of atoms of Apo and protein-ligand complex (Bisandrographolide and Andrographiside). (**b**) Root means square fluctuation of atoms of Apo protein and its complex with Bisandrographolide and Andrographiside. (**c**) Radius of gyration (RG) of Apo protein and its complex with Bisandrographolide and Andrographoside. (**d**) Hydrogen bond of the complex with Bisandrographolide and Andrographiside.

The root means square (RMSD) value indicates the stability of the protein-ligand complex during the simulation. The RMSD plot of 1DTP with andrographiside, bisandrographolide, and ampicillin, is represented in Figure 5a and shows an average value of 0.36 nm, 0.35 nm, and 0.33 nm, respectively. The complex was found to be stable after 20 ns, indicating the stability of the receptor-ligand complex during the simulation. Root mean square fluctuation (RMSF) indicates the mobility and stability of amino acid residues with a large degree of instability and immobility being indicated by large values of RMSF. The RMSF values of the complexes were found to be 0.166 nm, 0.160 nm, and 0.176 nm for andrographiside, bisandrographolide, and ampicillin, respectively, as shown in Figure 5b. The lower values for bisandrographolide and andrographiside show that these ligands are stable and rigid in the receptor during the whole MD simulation. The compactness, folding, and stability of the structure are revealed by the radius of gyration (RG), which, as depicted in Figure 5c, was found to have a steady average value of 1.7 nm. Bisandrographolide shows initial fluctuations at about 15 ns and is stable thereafter, and both bisandrographolide and andrographiside were found to show fewer fluctuations than the control drug ampicillin. This shows that the folding of ligands with 1DTP molecule is stable and compact. Hydrogen bonds, being the main stabilizing interactions between the ligand and the active site of the receptor, were analyzed and a plot of the number of hydrogen bonds participating in the interaction throughout the 100 ns simulation is depicted in Figure 5d. It was found that both bisandrographolide and andrographiside form stable hydrogen bonds in the active site of the apoprotein. A more accurate binding free energy between the receptor and the ligand can be calculated using the MM PBSA method. The binding free energy defines van der Waals energy, electrostatic energy, and polar solvation energy and was determined for andrographiside, bisandrographolide, and ampicillin and was found to be −124.505 ± 16.004 kJ/mol, −160.518 ± 14.849 kJ/mol, and −80.632 ± 13.621 kJ/mol, respectively.

The results show that bisandrographolide, having a more negative binding energy, has a higher affinity for the receptor protein, i.e., 1DTP. This can also be correlated with the lower RMSD value of bisandrographolide. Thus, with the MD simulation studies, it can be concluded that both the ligands, bisandrographolide and andrographiside, obtained from *A. paniculata* form a strong, stable, and energetically favorable interaction with the ligand binding site of the receptor molecule.

## 3. Materials and Methods

### 3.1. Selection and Preparation of Ligands

The 3D structures of 18 phytochemicals of *Andrographis paniculata* were downloaded from PubChem in SDF format. Hydrogen was added to the molecule and geometry optimization was done to obtain the most stable conformation using Avogadro software. The compounds were then saved in PDB format for further molecular docking studies. The 2D structures of the ligands are summarized in Table 5.

### 3.2. Selection and Preparation of Receptor Protein

**Figure 6:**
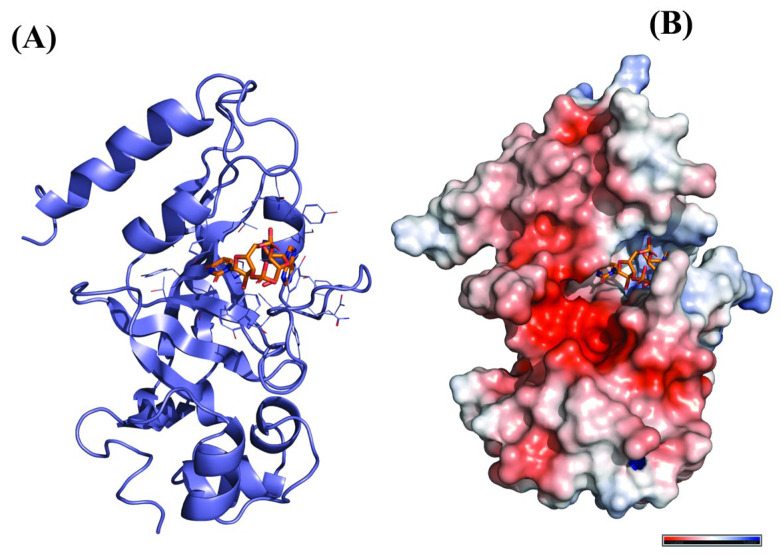
Three-dimensional Structure of Receptor, 1DTP. (**A**) Protein complexed with inhibitor molecules, (**B**) Optimized Protein (electrostatic view).

The catalytic domain of diphtheria toxin is used as a receptor for docking studies. The pdb structure (PDB ID:1DTP) with a resolution of 2.50 Å was retrieved from the RCSB PDB database. The receptor protein was optimized using Discovery studio software, where the inhibitor molecule (adenylyl (3′–5′) uridine 3′-monophosphate) and water molecules were removed and polar hydrogens were added for the final protein preparation. The final protein was saved in pdb format (Figure 6).

### 3.3. Analysis of Target Active Binding Sites

The active sites are the coordinates in the original target protein to which the drug molecule binds. These coordinates were analyzed using the active site prediction option of Discovery Studio Visualizer 2020. This software highlights the probable active site according to the co-crystallized structure, that is adenylyl (3′–5′) uridine 3′-monophosphate, and generates the grid file. The dimensions obtained after loading the protein were x = 0.013581, y = 4.689209, and z = 47.799581.

### 3.4. Molecular Docking

Molecular docking is executed in order to identify a bioactive compound from the cluster that can act as a potent drug against diphtheria toxin. The phytochemicals were docked against the target receptor using AutoDock vina, where ligands were presumed to be flexible, while the protein was considered to be rigid. The protein and ligand submitted in the PDBQT format are processed by AutoDock and the results are generated in the form of binding affinities. Different poses of ligands showing the best dockscore are obtained and visualized using Biovia Discovery studio in order to analyze their interaction with the receptor and illustrate the 2D poses that will reveal the type of interaction and the amino acids involved.

### 3.5. Pharmacodynamic Analysis and Toxicity Studies

The evaluation of drug-like characteristics of the ligands with the best dockscore and their ADMET analysis (Adsorption, Delivery, Metabolism, Excretion, Toxicity) were done using Swiss ADME (http://www.swissadme.ch/ accessed on 16 June 2021) and pkCSM (http://biosig.unimelb.edu.au/pkcsm/prediction accessed on 16 June 2021), respectively. This was performed in order to check the drug-likeness and fundamental physiological parameters such as TPSA (≤140 Å) and LogP (≤5) and also whether they follow Lipinski’s rule of five (Molecular weight ≤ 500, number of hydrogen bonds donors ≤ 5, number of hydrogen bond acceptors ≤ 10,). This is performed by submitting the ligands’ canonical SMILE format in the online tool. Further, the ADMET studies were done using pkCSM, which also accepts ligands in SMILE format and generates their physicochemical descriptors as well as ADMET parameters.

### 3.6. Molecular Dynamics Simulation

A detailed investigation of the virtual docking results can be done by molecular dynamics (MD) simulation studies. MD simulations 100 ns in duration were performed on the protein-ligand complex using GROMACS 2019.4. The force field coordinates for the protease and ligand were created using the PRODRG server and a simple point charge (SPC) water model was used to solvate the complex structures in a cubic periodic box (0.5 nm). The salt concentration of the complex system was maintained at 0.15 M by the addition of sufficient numbers of Na^+^ and Cl^−^ counter ions. NPT (No. of atoms, Pressure, Temperature) ensemble was applied for the NPT equilibration phase, after which MD simulation was executed for 100 ns. Once the 100 ns MD simulation was completed, the trajectories were used for different dynamics analyses such as the number of hydrogen bonds, root means square deviation (RMSD), root means square fluctuation (RMSF), and radius of gyration (Rg). The binding free energy of the inhibitor with protein over the simulation time was also calculated by utilizing the Molecular Mechanics/Poisson–Boltzmann Surface Area (MM/PBSA).

## 4. Conclusions

The discovery of novel compounds with potential biological activity and minimal-to-no adverse effects is a must in order to achieve effective diphtheria treatment. Hence, the present investigation demonstrates that the bioactive molecules of *Andrographis paniculata* might bepromising next-generation chemotherapeutic drugs and effectively used in the treatment of diphtheria. In the current study, we have used bioinformatics tools, Autodock vina, and GROMACS to identify and study the interaction of 18 phytoconstituents obtained from natural sources.Molecular docking studies have helped in the identification of potent molecules that can interact with 1DTP and a molecular dynamics study has validated the stable and strong interaction of the top dockscorers, namely bisandrographolide and andrographiside, with the protein, 1DTP. The results of the present study may serve as a basis for further optimization of the phytomolecules of *A. paniculate*, thereby establishing them as a reliable therapeutic against diphtheria.

## Figures and Tables

**Table 1:** Binding affinities of ligands.

Name of Ligands	Binding Affinity (Kcal/Mol)
7-methylwogonin	−8.5
Apigenin	−8.4
Luteolin	−7.6
Andrographidine C	−8.7
Andrographolide	−8.5
Neoandrographolide	−9.1
3-O-beta-D-Glucopyranosyl-14,19-dideoxyandrographolide	−8.7
14-deoxyandrographolide	−8.6
Andrograpanin	−8.5
Bisandrographolide	−10.4
Onysilin	−8.2
Andrographidin A	−8.4
14-Deoxy-12-Hydroxyandrographolide	−8.4
Andrographolactone	−8.6
8,17-Epoxy-14-Deoxyandrographolide	−8.5
Andrographiside	−9.5
14-Deoxy-11-Hydroxyandrographolide	−8.3
Isoandrographolide	−9.4
Ampicillin	−7.4

**Table 2:** Molecular interactions of best ligands.

Names of Ligands	Binding Affinity (Kcal/mol)	Amino Acids Involved in Interaction		
		Hydrogen Bond	Hydrophobic Interactions	Salt-Bridge Interactions
Neoandrographolide	−9.1	Tyr (2.68), His (2.74), Gly (1.88), Ser (1.98), Ser (2.41)	Tyr (3.1)	Absent
Bisandrographolide	−10.4	Gln (2.29), Thr (2.09), Asn (2.66)	Tyr (3.68), Ile (3.57), Pro (3.69), Thr (3.98), Tyr (3.66)	His (4.12), His (5.12), Lys (5.12)
Andrographiside	−9.5	Lys (2.38), Lys (2.89), Ser (2.93), Ser (2.15), Gly (2.75), Gly (2.44)	Tyr (3.75), Pro (3.33), Thr (3.89), Trp (3.72)	Absent
Isoandrographolide	−9.4	Gln (2.09), Asn (3.49)	Tyr (3.58), Ile (3.31), Pro (3.64), Trp (3.66)	His (5.35), Lys (4.88), Lys (2.86)
Ampicillin	−7.4	Lys (2.64), Lys (2.17), His (2.17), Asn (3.18)	Thr (3.59), Tyr (3.75), Tyr (3.71), Pro (3.88), Tyr (3.76)	Lys (4.19), Lys (4.12)

**Table 3:** Drug-like properties of ligands.

Name of Ligands	MW (g/mol)	#H-Bond Acceptors	#H-Bond Donors	TPSA (Å²)	iLOGP	ESOL Log S	Lipinski Violations	Lead Likeness Violations
7-methylwogonin	298.29	5	1	68.9	2.99	−4.12	0	0
Apigenin	270.24	5	3	90.9	1.89	−3.94	0	0
Luteolin	286.24	6	4	111.13	1.86	−3.71	0	0
Andrographidine C	460.43	10	4	148.05	2	−3.26	0	1
Andrographolide	350.45	5	3	86.99	2.45	−3.18	0	1
Neoandrographolide	480.59	8	4	125.68	3.27	−4.01	0	1
3-O-beta-D-Glucopyranosyl-14,19-dideoxyandrographolide	480.59	8	4	125.68	3	−4	0	1
14-deoxyandrographolide	334.45	4	2	66.76	2.91	−3.81	0	0
Andrograpanin	318.45	3	1	46.53	3.34	−4.21	0	1
Bisandrographolide	664.87	8	4	133.52	4.5	−7.26	1	3
Onysilin	300.31	5	1	64.99	2.88	−3.82	0	0
Andrographidin A	462.45	10	4	144.14	2.13	−3.01	0	1
14-Deoxy-12-Hydroxyandrographolide	350.45	5	3	86.99	2.61	−3.44	0	1
Andrographolactone	296.4	2	0	26.3	3.46	−4.66	1	1
8,17-Epoxy-14-Deoxyandrographolide	350.45	5	2	79.29	2.76	−3.26	0	1
Andrographiside	512.59	10	6	166.14	2.68	−2.63	2	1
14-Deoxy-11-Hydroxyandrographolide	350.45	5	3	86.99	2.87	−3.18	0	1
Isoandrographolide	350.45	5	2	75.99	2.67	−3.35	0	1
Ampicillin	349.40	5	3	138.03	1.14	−1.15	0	0

**Table 4:** ADMET properties of ligands.

Sr. No.	Name of Ligand	Absorption			Distribution	Metabolism							Excretion	Toxicity				
						Cyp												
						Substrate				Inhibitor								
		Water Solubility	Intestinal Absorption	Skin Permeability	Blood Brain Permeability	Cyp2d6	Cyp3a4	Cyp1a2	Cyp2c19	Cyp2c9	Cyp2d6	Cyp3a4	Total Clearance	Ames Toxicity	Herg	Max Tolerated Dose	Hepatotoxicity	Skin Sensitization
1	7-Methylwogonin	−4.12	High	−5.76	Yes	Yes	Yes	Yes	Yes	Yes	Yes	Yes	0.46	No	No/Yes	0.24	No	No
2	Apigenin	−3.94	High	−5.8	No	Yes	Yes	Yes	No	No	Yes	Yes	0.693	No	No/Yes	0.713	No	No
3	Luteolin	−3.71	High	−6.25	No	Yes	Yes	Yes	No	No	Yes	Yes	0.407	No	No/Yes	0.49	No	No
4	Andrographidine C	−3.26	Low	−8.43	No	No	Yes	No	No	No	No	Yes	0.55	No	No/Yes	0.801	No	No
5	Andrographolide	−3.18	High	−6.9	No	No	No	No	No	No	No	No	1.18	No	No	−0.10	No	No
6	Neoandrographolide	−4.01	High	−7.36	No	No	Yes	No	No	No	No	Yes	0.952	Yes	No	−0.436	No	No
7	3-O-Beta-D-Glucopyranosyl-14,19-Dideoxyandrographolide	−4	High	−7.46	No	No	Yes	No	No	No	No	Yes	0.91	No	No/Yes	−0.15	No	No
8	14-Deoxyandrographolide	−3.81	High	−5.9	Yes	No	No	No	No	No	No	No	−0.84	Yes	No	1.37	No	No
9	Andrograpanin	−4.21	High	−5.25	Yes	No	No	No	Yes	Yes	No	No	1.11	No	No	−0.61	No	No
10	Bisandrographolide	−7.26	Low	−6.04	No	No	No	No	No	No	No	No	0.225	No	No	−0.191	No	No
11	Onysilin	−3.82	High	−5.97	Yes	Yes	Yes	Yes	Yes	Yes	Yes	Yes	−30.9	Yes	No	0.438	No	No
12	Andrographidin A	−3.01	Low	−8.63	No	No	No	No	No	No	No	No	−32.9	Yes	No	0.438	No	No
13	14-Deoxy-12-Hydroxyandrographolide	−3.44	High	−6.53	No	No	No	No	No	No	No	No	1.2	No	No	0.02	No	No
14	Andrographolactone	−4.66	High	−4.76	Yes	No	No	No	No	Yes	No	No	1.2	No	No	0.2	No	Yes
15	8,17-Epoxy-14-Deoxyandrographolide	−3.26	High	−6.73	No	No	No	No	No	No	No	No	−0.43	Yes	No	1.39	No	No
16	Andrographiside	−2.63	Low	−9.41	No	No	No	No	No	No	No	No	1.033	Yes	No/Yes	−0.728	No	No
17	14-Deoxy-11-Hydroxyandrographolide	−3.18	High	−6.83	No	No	No	No	No	No	No	No	1.18	No	No	−0.4	No	No
18	Isoandrographolide	−3.18	High	−6.78	Yes	No	No	No	No	No	No	No	0.784	Yes	No	0.076	No	No
Control	Ampicillin	−2.577	High	−2.735	No	No	No	No	No	No	No	No	0.455	No	No	1.606	Yes	No

**Table 5:** 2D Structures of ligands.

Name of Ligand	Pubchem ID	Structure
7-methylwogonin	188316	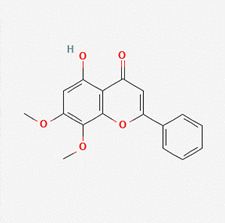
Apigenin	5280443	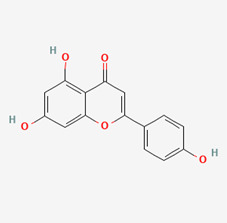
Luteolin	5280445	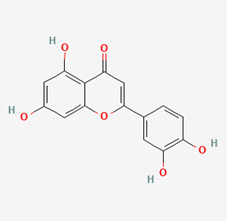
Andrographidine C	5318484	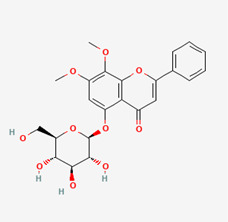
Andrographolide C	5318517	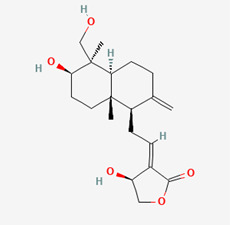
Neoandrographolide	9848024	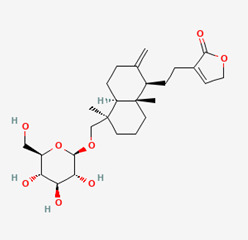
3-O-beta-D-Glucopyranosyl-14,19-dideoxyandrographolide	11576609	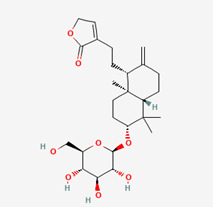
14-deoxyandrographolide	11624161	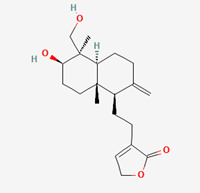
Andrograpanin	11666871	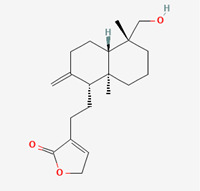
Bisandrographolide	12000062	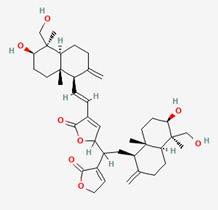
Onysilin	12041831	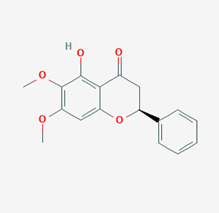
Andrographidin A	13963762	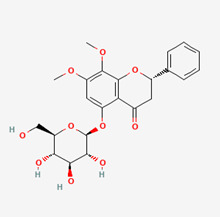
14-Deoxy-12-Hydroxyandrographolide	38350563	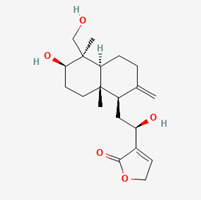
Andrographolactone	44206466	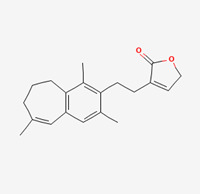
8,17-Epoxy-14-Deoxyandrographolide	44575263	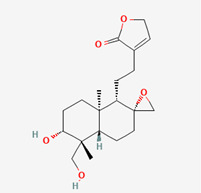
Andrographiside	44593583	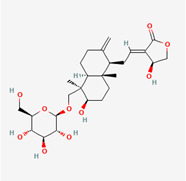
14-Deoxy-11-Hydroxyandrographolide	91884987	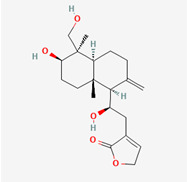
Isoandrographolide	101563019	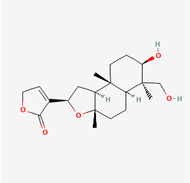
Ampicillin	6249	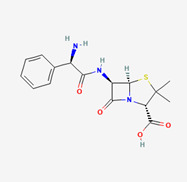

## Data Availability

The data presented in this study are available on request from the corresponding author.

## Sample Availability

Samples of the compounds are not available from the authors.
